# Acute effects of ingesting Java Fit™ energy extreme functional coffee on resting energy expenditure and hemodynamic responses in male and female coffee drinkers

**DOI:** 10.1186/1550-2783-4-10

**Published:** 2007-10-05

**Authors:** Lemuel W Taylor, Colin D Wilborn, Travis Harvey, Jennifer Wismann, Darryn S Willoughby

**Affiliations:** 1 Exercise Biochemistry Laboratory, Department of Health, Leisure, and Exercise Science, the University of West Florida, Pensacola, FL, USA; 2 Department of Exercise and Sport Science, University of Mary-Hardin Baylor, Belton, TX, USA; 3 Department of Physical Education Center for Physical Development Excellence, United States Military Academy, West Point, NY 10996 USA; 4 Exercise and Biochemical Nutrition Laboratory, Center for Exercise, Nutrition, and Preventive Health Research, Department of Health, Human Performance, and Recreation, Baylor University, Waco, TX, USA

## Abstract

**Background:**

The purpose of this study was to examine the effects of a functional coffee beverage containing additional caffeine, green tea extracts, niacin and garcinia cambogia to regular coffee to determine the effects on resting energy expenditure (REE) and hemodynamic variables.

**Methods:**

Subjects included five male (26 ± 2.1 y, 97.16 ± 10.05 kg, 183.89 ± 6.60 cm) and five female (28.8 ± 5.3 y, 142.2 ± 12.6 lbs) regular coffee drinkers. Subjects fasted for 10 hours and were assessed for 1 hour prior (PRE) and 3 hours following 1.5 cups of coffee ingestion [JavaFit™ Energy Extreme (JF) ~400 mg total caffeine; Folgers (F) ~200 mg total caffeine] in a double-blind, crossover design. REE, resting heart rate (RHR), and systolic (SBP) and diastolic (DBP) blood pressure was assessed at PRE and 1, 2, and 3-hours post coffee ingestion. Data were analyzed by three-factor repeated measures ANOVA (p < 0.05).

**Results:**

JF trial resulted in a significant main effect for REE (p < 0.01), SBP (p < 0.01), RER (p < 0.01), and VO_2 _(p < 0.01) compared to F, with no difference between trials on the RHR and DBP variables. A significant interaction for trial and time point (p < 0.05) was observed for the variable REE. The JF trial resulted in a significant overall mean increase in REE of 14.4% (males = 12.1%, females = 17.9%) over the observation period (p < 0.05), while the F trial produced an overall decrease in REE of 5.7%. SBP was significantly higher in the JF trial; however, there was no significant increase from PRE to 3-hours post.

**Conclusion:**

Results from this study suggest that JavaFit™ Energy Extreme coffee is more effective than Folgers regular caffeinated coffee at increasing REE in regular coffee drinkers for up to 3 hours following ingestion without any adverse hemodynamic effects.

## Background

The popularity of thermogenic supplements containing caffeine and other herbal products has increased over the past few years. In the U.S., approximately 110 million Americans drink coffee daily, while another 51 million are occasional coffee drinkers. Coffee generally contains caffeine in amounts ranging from 65–150 milligrams depending on the type of coffee and the method of preparation. Today, caffeine is appearing in many new products such as sports gels, energy drinks, and alcoholic beverages to provide a thermogenic effect in an effort to act as an ergogenic aid [1]. Even products that naturally contain caffeine are being altered to increase the amount of caffeine content. One of these products that have been introduced to the market (JavaFit™ Energy Extreme) is a new functional coffee beverage that contains 150 mg extra caffeine per serving, green tea extract, garcinia cambogia and niacin in an effort to produce a thermogenic product that could have added benefits over regular coffee. This new functional coffee is theorized to act as a thermogenic aid and possibly have implications on elevating the metabolic rate. Due to the popularity of daily coffee consumption, the rationale of altering an existing product that is used daily is sound.

Caffeine can have many effects in the body, but typically caffeine is thought of as a way to boost an individual's energy level on both a psychomotor level as well as a physiological level as an ergogenic aid in high-intensity and endurance exercise [2-5]. Caffeine has been identified as one of the very few purported ergogenic aids that has a significant effect on improving performance [1,6]. In addition, caffeine has been shown to stimulate metabolic rate in humans [7], so there could also be clinical implications for caffeine in the areas of weight loss and weight management.

Like other stimulants, caffeine has been advertised and sold as a way to stimulate energy expenditure and facilitate weight loss. Caffeine can stimulate both lipolysis and energy expenditure [8,9]. Caffeine ingestion has been studied alone and in conjunction with various herbal and vitamin products like ephedra, green tea extracts, calcium, tyrosine, chromium picolonate, capsaicin and garcinia cambogia. An earlier study on caffeine and energy expenditure indicated that a single dose of 100 mg of caffeine had a significant effect on resting metabolic rate (3–4% increase over 150 min), suggesting that caffeine can have a significant effect on energy balance at a commonly consumed dose, and possibly have positive effects in the treatment of obesity [10]. More recently, coffee ingestion (200 mg of caffeine) resulted in a 7% increase in energy expenditure for three hours following ingestion [11]. Recent research on the effects of caffeine supports the role of caffeine as a stimulus to increase total energy expenditure. A recent study found that caffeine alone increased energy expenditure by 13%, while doubling lipid turnover [8].

Several reports have examined the role of caffeine and coffee intake on a variety of diseases and health markers. It has been reported that coffee consumption has no significant effects on the risk of coronary artery disease [12,13] and may possibly even result in lower blood pressure on a chronic basis [13-15]. One safety marker that has been examined with caffeine or coffee intake is the effect on blood pressure. Despite the research supporting that chronic coffee intake could have a beneficial effect on blood pressure, research has shown that changes in systolic blood pressure are often observed following the acute ingestion of caffeine with the addition of other herbal products [16]. Many trials have shown this effect when caffeine has been combined with the herbal product ephedra [17,18]. A recent study also found increases in systolic blood pressure following acute coffee ingestion, but it should be noted that the combination of ingredients was different from the coffee blend examined in this study [16]. In addition, recent work has suggested that caffeine does have a stimulatory effect on systolic blood pressure, but the combination of other stimulants must be taken into consideration as well [19]. Thus, the stimulatory effects of caffeine on systolic blood pressure must be realized when ingesting caffeine and other stimulatory products.

Research has supported the valuable role of caffeine in metabolism and energy expenditure, and the safety of its consumption. One possible method of incorporating increased caffeine into daily consumption is to add it to daily use products (functional beverages and/or foods). The potential benefits could possibly include maintenance and possible reductions in body weight by increasing daily energy expenditure, which could have clinical implications with the prevalence of obesity in our society. This study is the first to examine the acute thermogenic responses between JavaFit™ Energy Extreme (JF) and a regular caffeinated coffee (F). Therefore, the specific purpose of this study was two-fold: 1) to assess the acute effects of ingesting a single dose of an additional caffeine-containing coffee beverage on resting energy expenditure for three hours after ingestion and 2) assess resting indices of hemodynamic function and general side effects for three hours after ingestion.

## Methods

### Subjects

Five physically active males (26 ± 2.1 y, 97.16 ± 10.05 kg, 183.89 ± 6.60 cm) and five females (28.8 ± 5.3 years, 64.64 ± 5.73 kg, 167.64 ± 7.37 cm) volunteered to participate in this study. Subjects were between the ages 18–35 and had to be regular coffee drinkers to participate in this study. A regular coffee drinker was designated as one that consumed at least 3 cups of coffee per week for at least one year prior to the study. Average self-reported daily caffeine intake for all subjects was 274.29 ± 53.22 milligrams per day. Subjects were not currently (or in the past 3 months) taking dietary supplements containing creatine, arginine, androstendione, thermogenics, or any other nutritional supplement. Subjects that were eligible were informed of the requirements of the study and signed an informed consent statement in compliance with the Institutional Review Board for the Protection of Human Subjects in Research guidelines of Baylor University.

### Study design

This study was conducted as a randomized, double blind, crossover design that was counterbalanced for the administration of two experimental trials. Subjects were familiarized one week prior to the start of testing. During the familiarization session, subjects completed a health history questionnaire, personal information sheet and signed an informed consent form. In addition, the subjects were verbally instructed on the study design and the requirements for each testing session. Subjects reported for first testing session on a 10-hour fast and were instructed to refrain from exercise and caffeine consumption 24 hours prior to each testing session. Subjects' height was assessed using standard anthropometry and total body weight was measured using a calibrated electronic scale with a precision of ± 0.02 kg (Bridgeview, Illinois). Subjects were then instructed to lie in a supine position for 1 hour. At the end of the 1-hour period, baseline resting energy expenditure (REE), heart rate (HR) and blood pressure (SBP and DBP) were assessed. Following the baseline assessment, subjects were randomly assigned to drink 1.5 cups (354 milliliters) of either JavaFit™ Energy Extreme (JF) coffee [~400 mg of total caffeine, 600 mg green tea extracts (EGCG), <15 mg of garcinia cambogia and ~30 mg of niacin] or Folgers Regular (F) coffee [~200 mg of caffeine]. The coffee was prepared using 65 milligrams of the respective coffee with 354 milliliters of distilled water using standard drip-brewed methods 20 minutes prior to the start of each individual trial. Subjects were required to ingest the entire amount of black coffee in 15 minutes. Following ingestion, subjects rested in a supine position for 3 hours in which REE, HR, SBP and DBP were assessed in the last 20 minutes of each observation hour following the ingestion of the coffee. Following the 3-hour assessment period, subjects completed a side effects questionnaire to assess any possible adverse reactions from the coffee ingestion. Subjects reported to the lab one week later for the second session of the crossover design using the other coffee and repeated the 4-hour testing protocol.

### REE and hemodynamic assessment

Resting energy expenditure was assessed using a Parvo Medics' TrueOne^® ^2400 (ParvoMedics, Sandy, UT) integrated metabolic measurement system. Metabolic carts were calibrated everyday 30 minutes prior to the beginning of testing. Resting energy expenditure was measured in a supine position and REE values were calculated during the final 10 minutes of each 1-hour session to ensure each subject had stabilized to a resting state. Resting heart rate was assessed in the supine position with wireless Polar heart rate monitors (Polar Electro Inc., Lake Success, NY) using standard procedures at the end of each observation hour. Resting blood pressure was assessed in the supine position using a mercurial sphygmomanometer using standard procedures at the end of each observation hour.

### Dietary records

The subjects' diets were not standardized and subjects were asked not to change their dietary habits during the course of the study with the exception of limiting their caffeine intake 24 hours prior to each testing session. However, subjects were required to keep dietary records for the 24 hours prior to each testing session of the crossover design to evaluate their daily macronutrient intake for carbohydrate, fat, protein and total calories. The dietary records were evaluated with the Food Processor dietary assessment software program (ESHA Research Inc., Salem, OR).

### Side effects assessment

The possible side effects of the two coffees were assessed with a Side Effects Questionnaire (Table 1). Variables that were addressed included: dizziness, headache(s), fast or racing heart rate, heart skipping or palpitations, shortness of breath, nervousness, blurred vision, as well as a self-report of any other unusual or adverse effects. The questionnaire assessed both the frequency of occurrence for each variable, as well as severity of these variables at the time the questionnaire.

**Table 1:** Side-effects questionnaire that was administered following each coffee trial.

Testing Session	Baseline	T1	T2
Did you consume all the coffee?			
Rate the frequency of the following symptoms according to the scale where:			
0 = none			
1 = minimal			
2 = slight			
3 = occasional			
4 = frequent			
5 = severe			
Dizziness?			
Headache?			
Fast or racing heart rate?			
Heart skipping or palpitations?			
Shortness of breath?			
Nervousness?			
Blurred Vision?			
Any other unusual or adverse effects?			
Rate the severity of the following symptoms according to the scale where:			
0 = none			
1 = minimal			
2 = slight			
3 = moderate			
4 = severe			
5 = very severe			
Dizziness?			
Headache?			
Fast or racing heart rate?			
Heart skipping or palpitations?			
Shortness of breath?			
Nervousness?			
Blurred Vision?			
Any other unusual or adverse effects?			

### Statistical analysis

Dependent variables were analyzed by utilizing separate three-factor [coffee (F or JF) × gender (M or F) × time point (PRE & 1, 2, 3 hours post-ingestion)] factorial analyses of variance (ANOVA) with repeated measures for each criterion variable. In addition, the within trial changes for REE were analyzed with dependent t-tests. Data obtained from the Side Effects Questionnaire were analyzed with separate ANOVA's for each respective variable. Significant between-group differences were determined involving the Neuman-Keuls Post Hoc Test. All statistical procedures were performed using SPSS 11.0 software and a probability level of < 0.05 was adopted throughout. All data are reported as means ± standard deviation.

## Results

### Energy expenditure measures

**Figure 1:**
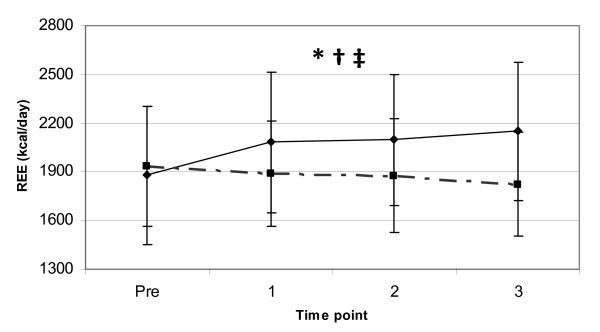
**REE response to coffee ingestion**. Change in supine resting energy expenditure (mean ± SD) between two trials (broken line = F, solid line = JF) from pre to 3-hours following coffee ingestion. * = significant main effect for trial (p < 0.05). † = a significant main effect for gender (p < 0.01). ‡ = a significant interaction for trial and time point (p < 0.05).

The REE responses between the two coffee trials indicated that there was a significantly higher elevation in REE over the 3-hour observation period during the JF trial. Significant main effects for trial (p < 0.01) and gender (p < 0.01) for the variable REE were observed indicating that the JF trial and the males had an overall higher REE. In addition, a significant interaction for trial and time point (p < 0.05) was observed for the variable REE. Dependent t-tests supported these findings indicating a significant increase in REE from PRE to 3-hours post (p < 0.01) in the JF trial, while the F trial showed a trend (p = 0.058) for a significant decrease in REE from PRE to 3-hours post. Mean values at PRE and 3-hours post for the variable REE were 1,932.83 ± 370.25 and 1,822.91 ± 323.09 kcal for the F trial and 1,858.23 ± 412.89 and 2179.75 ± 424.34 kcal for the JF trial (see Figure 1), respectively. This change in REE from PRE to 3-hours post represents an overall average increase in REE for both genders of 14.4% (males = 12.1%, females = 17.9%) in the JF trial, whereas a decrease in REE of 5.7% from the PRE to 3-hour post observation period was observed in the F trial.

**Figure 2:**
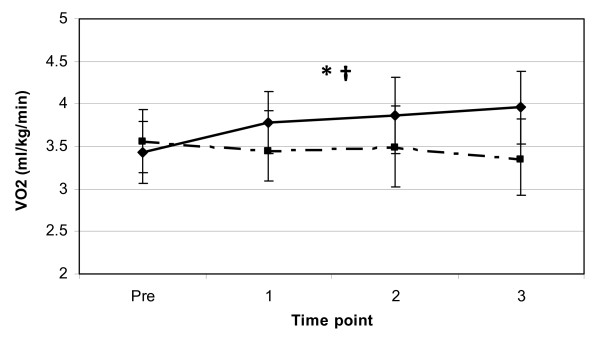
**Resting VO_2 _response to coffee ingestion**. Change in supine resting VO_2 _(mean ± SD) between two trials (broken line = F, solid line = JF) from pre to 3-hours post coffee ingestion. * = significant main effect for trial (p < 0.05). † = a significant main effect for gender (p < 0.01).

In accordance with the differences observed in REE, significant main effects for trial (p < 0.01) and gender (p < 0.01) were observed for resting VO_2 _indicating that the JF trial and that the females had a higher resting VO_2_, with no significant differences for time point or interaction effects. Overall mean values at PRE and 3-hours post for the variable VO_2 _were 3.55 ± 0.39 and 3.35 ± 0.47 ml/kg/min for the F trial and 3.44 ± 0.36 and 3.96 ± 0.44 ml/kg/min for the JF trial (see Figure 2), respectively. These results show a 15.1% elevation in resting VO_2 _from PRE to 3-hours post in the JF trial, while the resting VO_2 _in the F trial decreased 5.6% over the 3-hour observation period.

**Figure 3:**
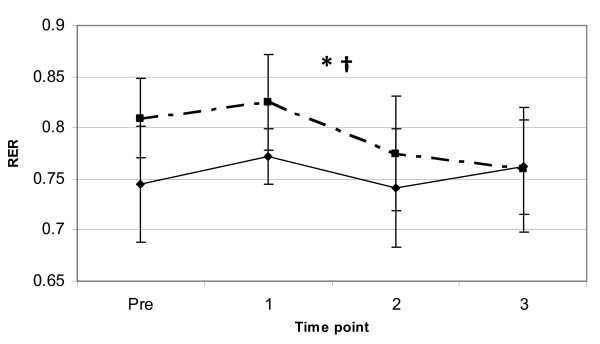
**RER response to coffee ingestion**. Change in supine resting RER (mean ± SD) between two trials (broken line = F, solid line = JF) from pre to 3-hours post coffee ingestion. * = significant main effect for trial (p < 0.05). † = a significant main effect for gender (p < 0.01).

As for the variable resting RER, a significant main effect for resting RER (p < 0.01) was observed indicating that the JF trial had an overall lower RER, with no significant differences for time point, gender or interaction effects. Overall mean values at PRE and 3-hours post for the variable RER were 0.809 ± 0.056 and 0.759 ± 0.046 for the F trial and 0.745 ± 0.039 and 0.762 ± 0.061 for the JF trial (see Figure 3), respectively. These results represent a 2.3% elevation in resting RER from PRE to 3-hours post in the JF trial, while the resting RER in the F trial decreased 6.2% over that 3-hour observation period.

### Hemodynamic measures

**Figure 4:**
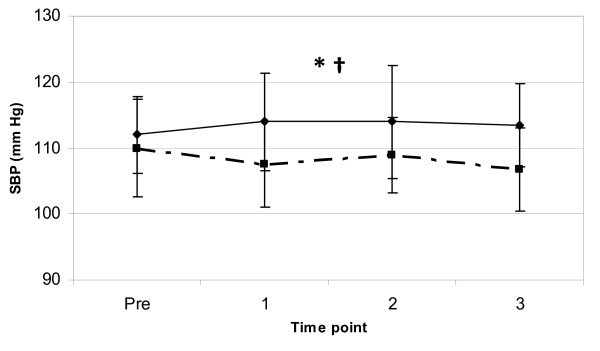
**SBP response to coffee ingestion**. Change in supine resting SBP (mean ± SD) between two trials (broken line = F, solid line = JF) from pre to 3-hours post coffee ingestion. * = significant main effect for trial (p < 0.05). † = a significant main effect for gender (p < 0.01).

Hemodynamic markers were observed to examine the effect of the two coffee trials. Significant main effects for trial (p < 0.01) and gender (p < 0.01) were observed for resting SBP indicating that the JF trial had an overall higher SBP and the females had a lower SBP than males, with no significant differences for time point or interaction effects. Mean values at PRE and 3-hours post for the variable SBP were 110.0 ± 7.48 and 106.8 ± 6.33 for the F trial and 112.0 ± 5.8 and 113.4 ± 6.33 for the JF trial (see Figure 4), respectively. SBP was significantly higher in the JF trial, however the ingestion of the JF coffee did not significantly increase SBP over time during the PRE to 3-hour post observation period (p = 0.61). These results show a 1.3% elevation in resting SBP from PRE to 3-hours post in the JF trial, while the resting SBP in the F trial decreased 2.9% over that 3-hour observation period.

**Figure 5:**
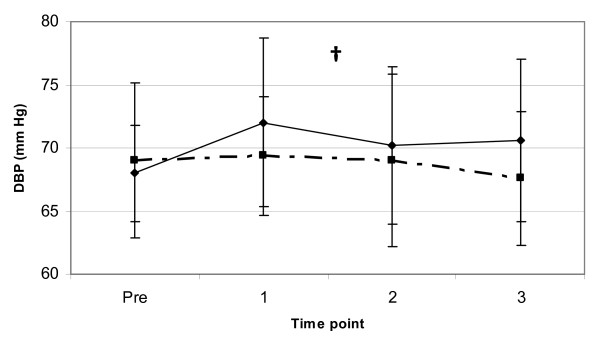
**DBP response to coffee ingestion**. Change in supine resting DBP (mean ± SD) between two trials (broken line = F, solid line = JF) from pre to 3-hours post coffee ingestion. † = a significant main effect for gender (p < 0.01).

**Figure 6:**
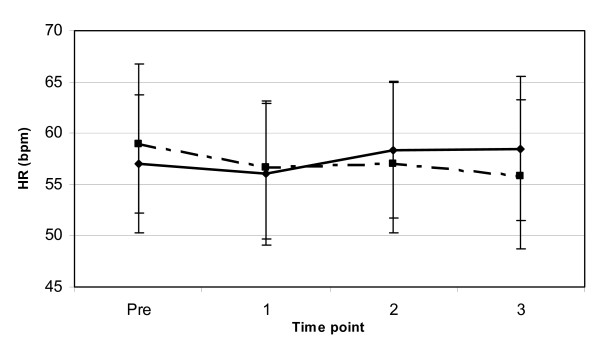
**RHR response to coffee ingestion**. Change in supine resting RHR (mean ± SD) between two trials (broken line = F, solid line = JF) from pre to 3-hours post coffee ingestion.

Additional hemodynamic markers assessed in this study included HR and DBP. A significant main effect for gender (p < 0.01) was observed for resting DBP indicating that the females had a lower resting DBP than the males, with no significant differences for time point or interaction effects (see Figure 5). The results of this study indicated that there were no significant interactions or main effects for trial, gender or time (p > 0.05) for resting HR (see Figure 6). These data suggest that the consumption of both F and JF have no affect on resting HR and DBP for 3-hours following ingestion.

### Nutritional analysis

No significant changes in 24-hour dietary recalls were observed between subjects for macronutrient content of protein (g), carbohydrates (g), fat (g) and total caloric intake (kcal) (see Table 2), or caffeine ingestion prior to each respective trial (F or JF).

**Table 2:** Self-reported 24-hour dietary recall analysis (mean ± SD) between two trials.

**Variable**	**F**	**JF**	**Significance**
Protein (g)	169 ± 57	171 ± 59	p = 0.998
Fat (g)	56 ± 18	54 ± 20	p = 0.959
Carbohydrates (g)	326 ± 108	319 ± 112	p = 0.939
Total Calories (kcal)	2,478 ± 841	2,458 ± 828	p = 0.96

Data are means and ± standard deviations

### Side effects

Subjects completed a Side Effects Questionnaire following both 4-hour testing sessions to assess any potential side effects of the coffee (caffeine) ingestion (Table 1). No significant differences were observed for the frequency of occurrence or for the severity of the side effects dizziness, headache, fast or racing heart rate, heart skipping or palpitations, shortness of breath, nervousness and blurred vision. One female subject reported light-headedness at the end of the 3-hour observation period, but this was reported following both testing sessions of the crossover design, and was more likely due to the duration of the fasting condition associated with each session.

## Discussion

The major findings of this study suggest that drinking coffee containing approximately 400 mg total caffeine (an additional 150 mg per serving compared to regular, caffeinated coffee), green tea extract, garcinia cambogia and niacin does have significant effects on resting energy expenditure compared to normal caffeinated coffee for 3-hours following ingestion in healthy and physically active, regular coffee drinkers. The significant difference in REE between the two trials occurred with a slight difference in SBP, while resting HR and DBP were not affected by the addition of 200 mg of caffeine over regular, caffeinated coffee, as well as the additional herbal ingredients that are present in the experimental coffee (JF).

Previous research has supported the notion that acute caffeine and/or coffee ingestion can have significant effects on REE. These previous findings have shown increases in REE over 2 to 3-hour time periods following ingestion and have utilized various combinations of caffeine combined with additional ingredients including ephedra, black tea, garcinia cambogia, citrus aurantium and chromium polynicotinate [16,20,21]. Caffeine has been the common ingredient in all of these studies and caffeine alone has been demonstrated to affect REE in human studies [7,8,10,11]. This is important because another previously studied ingredient (ephedra) that has been combined with caffeine is not overly available on the market and now other ingredients are being combined with caffeine to determine if they can alter the metabolic rate following ingestion. This study examined a functional coffee beverage that has 150 mg of additional caffeine per serving as well as an herbal combination of green tea extracts, garcinia cambogia and niacin. This is the first study to examine the acute changes in metabolic rate using this type of functional coffee. Our findings suggesting that consumption of JF can have significant effects on REE are in agreement with previous research indicating that caffeine, coffee and green tea have been shown to have positive effects on REE in humans [7,8,10,11,16,22]. Knowing the effects that caffeine and green tea extracts can have on the metabolic rate, the increases in REE observed in this study are likely a result of adding additional caffeine and green tea extracts that were not present in the regular coffee trial. In addition, the relatively small amounts of garcinia cambogia and niacin are probably not significant enough to affect any of the variables measured in this study. Garcinia cambogia has been studied as a possible means of increasing metabolism and promoting weight loss, but research has not supported these proposed benefits [23].

In the current study, the JF trial had a significantly lower RER when compared to the F trial. However, no significant changes were observed over time for RER, thus these findings suggest that fuel oxidation was not significantly affected by the experimental coffee trial. Our findings do not agree with a recent study that utilized a similar coffee blend containing additional caffeine, citrus aurantium and chromium polynicotinate which reported a significantly higher RER in the experimental coffee over placebo [16]. These differences are most likely attributed to the differences in the added ingredients to the coffee blend (i.e., green tea extracts and niacin vs. citrus aurantium and chromium polynicotinate).

The alterations in REE and RER were observed in the presence of a significantly higher resting VO_2 _in the JF trial. Resting VO_2 _increased 15.1% from PRE to 3-hour post time point, while the VO_2 _of the F trial decreased 5.6% over the same time period. Despite these changes, the interaction effect for trial and time point for the variable VO_2 _only showed a trend for significance (p = 0.06), thus we cannot suggest that the JF trial elicited a higher VO_2 _response over the 3-hour observation period. However, these findings in conjunction with the significant elevations in REE do suggest that ingestion during the JF trial positively affects oxidative metabolism in the body in comparison to the F trial.

An increase in SBP is an occurrence that has previously been associated with the ingestion of products containing caffeine and/or herbal ingredients [16,18,19]. The findings of this study found a significantly higher SBP in the JF trial. Despite the significant difference, the small increase over time (1.3%) was not a significant elevation. It should be noted that the JF coffee compared to the regular coffee (F) did have approximately 150 mg more caffeine per serving, and the F trial did demonstrate a decrease (2.9%) in SBP over the same time period. It is likely that the additional caffeine and possibly the green tea extracts contained in the JF coffee resulted in the small sustained increases in SBP over time. In addition to the SBP responses, the hemodynamic variables resting HR and DBP were not affected by either of the coffees, which are in agreement with previous research [16,24].

Caffeine consumption has traditionally been associated with other issues concerning health; however the claims seem to not be supported. As for blood pressure, caffeine consumption has been linked to acute increases in blood pressure, but habitual coffee consumption does not seem have adverse effects on blood pressure [15]. However, some studies do suggest that individuals that already have existing high blood pressure issues may benefit from reducing or restricting coffee intake in these populations [25,26]. Overall, habitual coffee consumption does not seem to lead to negative effects on blood pressure unless there is a pre-existing problem with high blood pressure. Diabetes is another area of health that has been studied in its relationship to caffeine/coffee consumption. Studies suggest that coffee drinking is associated with a higher insulin sensitivity and a lower risk of type 2 diabetes [27-31], and that total caffeine intake from all sources was associated with a significantly lower risk for diabetes in men and women [29]. In addition, various studies have found no negative relationship between caffeine, coffee or tea consumption with the following types of cancer: breast cancer [32], oral/pharyngeal and esophageal cancer [33], colon cancer [34], epithelial ovarian cancer [35], liver cancer [36], thyroid cancer [37] and pancreatic cancer [38] in various populations. Thus, the overall health implications with caffeine and coffee consumption do not seem to have a negative effect on health and the possible positive effects on REE and metabolism show potential in the areas of weight management and weight loss.

## Conclusion

The major findings of this study indicate that the consumption of 1.5 cups of a functional coffee beverage containing approximately 400 mg caffeine (150 mg additional caffeine per serving compared to regular, caffeinated coffee), green tea extracts, garcinia cambogia and niacin can significantly increase the resting energy expenditure over a 3-hour time period in comparison to regular caffeinated coffee. These findings could have potential clinical implications in the area of weight maintenance and weight loss. However, the implications of caffeine intake on other processes in the body must not be discounted and should be taken into consideration before this is considered a possible path for improving weight maintenance and weight loss. Despite the overall difference between trials, SBP was not affected over time with the additional caffeine consumption present in the JF coffee. Resting HR and DBP were not affected with ingestion of either of the two coffees and both coffees were well tolerated with no significant reported side effects following ingestion. Future research needs to be conducted to examine the chronic effects of this product to see if it has implications in weight loss and/or weight maintenance resulting from the acute increases in resting energy expenditure.

## Competing interests

The author(s) declare that they have no competing interests.

## Authors' contributions

LWT participated in the design of the study, coordination and data acquisition, performed the statistical analysis, and drafted the manuscript. CDW participated in the data acquisition, helped in statistical analysis, and helped to draft the manuscript. TH participated in data acquisition and helped to draft the manuscript. JW participated in data acquisition and helped draft the manuscript. DSW conceived the study, developed the study design, secured the funding for the project, helped with statistical analysis, and helped draft the manuscript. All authors read and approved the final manuscript.
